# Inherited thrombophilia in a Han Chinese family caused by prothrombin Ile441Met mutation

**DOI:** 10.1016/j.rpth.2025.102987

**Published:** 2025-07-29

**Authors:** Si-Yuan Wen, Fei-Fei Chen, Ji-De Chen, Pan Tao, Chi Meng, Jing Huang, Xin Kang, Wei Chen, Chang-Qing Zhou

**Affiliations:** 1 Department of Neurology, Bishan Hospital of Chongqing Medical University, Chongqing, China; 2 Department of Clinical Laboratory, Bishan Hospital of Chongqing Medical University, Chongqing, China

**Keywords:** F2 gene, inherited thrombophilia, intracranial venous sinus thrombosis, prothrombin, venous thrombosis embolism

## Abstract

**Background:**

Inherited thrombophilia (IT) is a genetically determined predisposition to thromboembolic events. Beyond the well-known G20210A mutation, there has been limited research on other prothrombin mutations in the Chinese population.

**Objectives:**

This study aimed to identify and characterize a novel prothrombin mutation in a Han Chinese family with IT.

**Methods:**

Clinical information was collected from the proband and his related family members. Coagulation tests, including protein S, plasminogen, protein C, and antithrombin Ⅲ activities, were conducted. Whole-genome sequencing was conducted on the proband and his mother to identify the causative mutation, and suspected mutations were verified in other family members using whole-exon sequencing. Thrombin generation assay was performed to evaluate hypercoagulable states.

**Results:**

Among the 53 family members, 11 individuals had a history of venous thromboembolism (VTE). Genetic analysis of 9 family members identified a novel heterozygous prothrombin mutation, p.Ile441Met (c.1323A>G), in 6 individuals with VTE history. These mutation carriers exhibited various forms of VTE, predominantly pulmonary embolism and lower-limb deep vein thrombosis. Routine coagulation tests showed no significant abnormalities in prothrombin time and activated partial thromboplastin time, while 5 carriers exhibited decreased protein S activity. Thrombin generation assay revealed a hypercoagulable state, characterized by shortened lag time, increased thrombin peak, and elevated endogenous thrombin potential.

**Conclusion:**

The Ile441Met mutation is a novel prothrombin mutation associated with IT in the Han Chinese population, which induces a hypercoagulable state, leading to various forms of VTE. Further studies are needed to validate these findings and investigate the underlying pathogenic mechanisms.

## Essentials

• A novel prothrombin mutation, Ile441Met, is identified in a Han Chinese family.
• All the mutation carriers experienced at least 1 venous thromboembolism event.
• Clinical information, coagulation tests, and thrombin generation assay were presented.
• TGA results indicate a hypercoagulable state in the carriers.

## 1 Introduction

Inherited thrombophilia (IT) can cause various forms of venous thromboembolism (VTE), including pulmonary embolism (PE), lower-limb deep vein thrombosis (DVT), mesenteric vein thrombosis, and cerebral venous sinus thrombosis (CVST) [1]. The incidence of VTE is gradually increasing worldwide, with the overall annual incidence among populations in China (10-30/100,000) being lower than that in Europe and the United States (75-269/100,000) [2]. IT is commonly associated with mutations in genes encoding physiological anticoagulants, such as antithrombin (AT), protein C (PC), and protein S (PS), which result in the loss of protein anticoagulant function, or with mutations in procoagulant proteins, such as *F5* Leiden and *F2* G20210A mutations, which enhance protein procoagulant activity [3]. There are significant racial differences in the genetic etiology of IT, with the *F5* mutation being most prevalent in Caucasian IT families (38.2%), while the *PROS1* mutation is most common in East Asian IT families (40.4%) [2].

Prothrombin, also known as coagulation factor Ⅱ, is a plasma glycoprotein encoded by the *F2* gene. During coagulation, prothrombin is activated to thrombin, an enzyme that plays a central role in blood coagulation [4]. Mutations in the *F2* gene often lead to prothrombin deficiency, which is typically manifested as a bleeding tendency [5]. However, some heterozygous mutations in the *F2* gene may lead to atypical thrombosis [6]. Although the G20210A mutation is the most common *F2* gene mutation associated with IT, it is more prevalent in the Caucasian population and relatively rare among the Han Chinese population [7,8]. The G20210A mutation is associated with a significant increase in prothrombin levels, making it a moderate risk factor for VTE [9]. Due to its rarity in China, there is limited investigation into the *F2* gene mutations, aside from G20210A, which contribute to thrombophilia in the Chinese population.

In this study, we identified a novel prothrombin mutation, p.Ile441Met (c.1323A>G), in a Han Chinese family, where multiple members presented with VTE. This mutation has not been previously reported, suggesting a potentially novel genetic variant contributing to thrombophilia. The aim of this study was to investigate the clinical and genetic characteristics of the prothrombin Ile441Met mutation in relation to IT, providing new insights that may support improved diagnosis and management of thrombophilia in the Chinese population.

## 2 Methods

### 2.1 Clinical data collection

Clinical data on the thrombophilia family members were collected from the proband (Ⅲ-9) and related family members, including detailed thrombotic and hemorrhagic history, comorbidities, medication history, and VTE risk factors before onset. The American Society of Hematology 2020 guidelines for management of VTE were used to assess VTE risk factors [10]. Additionally, Ⅲ-9 and available family members underwent testing for complete blood count, liver function, renal function, blood lipids, blood glucose, and antinuclear antibody spectrum to screen for other conditions that could influence coagulation function. All participants voluntarily participated in this study at Bishan Hospital of Chongqing Medical University, between September 2023 and October 2024. The study was approved by the ethics committee of Bishan Hospital of Chongqing Medical University (approval number: cqbykyll-20230427-01), and informed consent was obtained from all participants.

### 2.2 Coagulation tests

Peripheral venous blood samples were collected from the available family members using anticoagulant tubes containing 109 mmol/L sodium citrate. After centrifugation, the upper plasma layer of the blood samples was used for routine coagulation tests and additional analysis. Routine coagulation tests, including prothrombin time (PT), activated partial thromboplastin time (aPTT), thrombin time, prothrombin activity (PTA), international normalized ratio, fibrinogen, and D-dimer, were performed using the Sysmex CS-5100 automatic hemostasis analyzer (Siemens Healthcare Diagnostics). PS activity and lupus anticoagulant were measured using the coagulation method, and plasminogen, PC, and AT Ⅲ activities were measured using the chromogenic substrate method (KingMed Diagnostics). For PS activity testing, all samples were analyzed using Innovance Protein S Kit (Siemens Healthcare Diagnostics) on the Sysmex CS-5100, which measures prolongation of aPTT in the presence of excess activated PC.

### 2.3 *F2* gene analysis

Whole-genome sequencing was conducted on Ⅲ-9 and his mother (Ⅱ-7) to identify the causative mutation for IT in the family, and the suspected mutations were verified in other members using whole-exon sequencing. Genetic sequencing of Ⅲ-9 and available family members was conducted on the Illumina sequencing platform (Illumina Inc). The obtained data exhibited an average sequencing depth of ≥90× of known gene exons and their upstream and downstream 5 bp sequences in the human genome, with approximately 98% of the target sequences achieving a sequencing depth exceeding 20×. Base calling was conducted on all sequenced fragments, and data analysis was carried out using the Genome Analysis Toolkit software suite. Sequence alignment was performed using Burrows–Wheeler Aligner Tool, mapped to the University of California Santa Cruz hg19 reference genome. Variants were annotated with Variant Effect Predictor software (the European Molecular Biology Laboratory's European Bioinformatics Institute), and variant screening was conducted using genetic disease databases, variant databases, and population databases, including ClinVar, Online Mendelian Inheritance in Man, the Human Gene Mutation Database, and the Genome Aggregation Database. DNA analysis procedures were established and validated by KingMed Diagnostics.

### 2.4 Thrombin generation assay

Thrombin generation assays (TGAs) were performed using platelet-poor plasma from family members carrying the Ile441Met mutation who had not used any medications that could affect coagulation status within the past 3 months. Healthy subjects matched for gender and age were selected as normal controls. The main parameters included lag time, time to peak, peak height, velocity index, and endogenous thrombin potential (ETP), providing a comprehensive profile of thrombin generation dynamics in each subject. TGA was assessed using Reagent C Low with a fluorogenic substrate on the Ceveron s100 calibrated automated thrombography and coagulation analyzer (all from Technoclone Herstellung von Diagnostika und Arzneimitteln GmbH) by Thalys MedLab.

### 2.5 Protein function prediction

Multiple online tools, including Sorting Intolerant From Tolerant (SIFT, https://sift.bii.a-star.edu.sg/), PolyPhen-2 (http://genetics.bwh.harvard.edu/pph2/), and MutationTaster (https://mutationtaster.org/), were used for evaluating and predicting the pathogenicity of the Ile441Met mutation and its impact on protein function. The amino acid sequences of prothrombin in humans and several other species were collected from the National Center for Biotechnology Information protein database, and ClustalW 2.1 software (the Conway Institute of University College Dublin) was used to compare the sequences surrounding Ile441 to evaluate the conservation of this site. Homology modeling was performed using SWISS-MODEL (https://swissmodel.expasy.org/) to assess the protein structure of mutant prothrombin.

### 2.6 Statistical analysis

IBM SPSS Statistics 29.0 software was used for statistical analysis. Descriptive statistics were used to summarize the clinical characteristics of the thrombophilia family members. The mean, maximum, and minimum values were calculated for continuous variables, while frequencies and percentages were calculated for categorical variables.

## 3 Results

### 3.1 Clinical phenotype

Ⅲ-9, a 38-year-old Han Chinese male, was admitted to the hospital due to progressive headache with weakness in the lower extremities for 4 days. He was diagnosed with acute ischemic stroke (AIS) through imaging examinations 12 years ago, and only mild dysarthria remained when he was discharged. During hospitalization, he exhibited sudden dyspnea and recurrent episodes of altered consciousness accompanied by limb convulsions. Brain computed tomography angiography indicated bilateral parietal lobe hemorrhage, along with reduced enhancement of the superior and inferior sagittal sinuses, straight sinus, transverse sinuses, and sigmoid sinus. Digital subtraction angiography further confirmed the occlusion of the superior sagittal sinus, inferior sagittal sinus, straight sinus, and confluence of sinuses. Pulmonary computed tomography angiography indicated the presence of emboli in both the left and right main pulmonary arteries, as well as multiple pulmonary emboli in the right middle lobe, lower lobe, lingula, and lower lobe of the left lung. Based on the clinical manifestations and imaging examination results, the patient was diagnosed with CVST, PE, and secondary epilepsy. After receiving sequential anticoagulation therapy with enoxaparin and warfarin, the patient was discharged from hospital with only mild symptoms of unclear speech.

**Figure 1:**
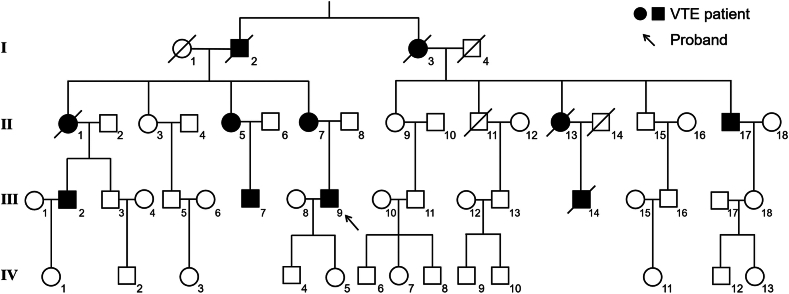
The pedigree of the family. VTE, venous thromboembolism.

Among the 53 members of the extended family of Ⅲ-9, 11 individuals have a history of VTE. The family pedigree is shown in Figure 1. Ⅱ-7 has a history of DVT in both lower limbs and mesenteric vein thrombosis. Unfortunately, Ⅱ-7 had mesenteric venous thrombosis during this study, which occurred not long after participating in general blood and coagulation tests and before participating in TGA. She was transferred to another hospital for intestinal resection, after which rivaroxaban was administered for anticoagulation therapy. Ⅲ-9’s uncle (Ⅱ-17) and older male cousins (Ⅲ-2 and Ⅲ-7) have a history of lower-limb DVT and PE. Ⅲ-9’s aunt (Ⅱ-5) has a history of lower-limb DVT, while another aunt (Ⅱ-1) died of AIS. Ⅲ-9’s younger male cousin (Ⅲ-14) died of mesenteric vein thrombosis. The cause of death of the family members Ⅰ-2, Ⅰ-3, and Ⅱ-13 was not directly attributable to VTE-related diseases; however, they had a prior history of lower-limb DVT. No thrombotic event was found in other existing family members.

Among all the family members, 9 individuals (Ⅱ-5, Ⅱ-7, Ⅱ-15, Ⅱ-17, Ⅲ-2, Ⅲ-7, Ⅲ-9, Ⅲ-18, and Ⅳ-4) were available and participated in this study. Clinical information for these family members is provided in Table 1. A total of 6 individuals (66.67%) have a history of VTE, consisting of 2 females and 4 males, with an average age of onset of the first VTE event of 35.5 years (range, 21-53 years). Only Ⅱ-7 had a significant risk factor for pregnancy prior to the initial onset of VTE. There were no significant abnormalities in the complete blood count, liver function, renal function, blood lipids, or blood glucose of any family member, and their antinuclear antibodies were all negative.

**Table 1:** Clinical information of the family members.

Members	Sex	Age (y)	BMI	Mutation carrier	Site of thrombosis (age of onset [y])	Significant risk factor	Concomitant disease	Anticoagulant therapy
**Ⅱ-5**	F	67	24.30	Y	DVT (53)	None	HT, HL	None
**Ⅱ-7**	F	59	23.26	Y	DVT (21), MVT (59)	Pregnancy	HT	Rivaroxaban (20 mg, every day)
**Ⅱ-17**	M	53	27.68	Y	DVT (43), PE (52)	None	None	Rivaroxaban (10 mg, every day)
**Ⅲ-2**	M	51	27.76	Y	DVT (45), PE (45)	None	None	Rivaroxaban (irregular)
**Ⅲ-7**	M	42	26.99	Y	DVT (27), PE (27)	None	None	Warfarin (1.25 mg, every day)
**Ⅲ-9**	M	38	25.83	Y	AIS (24), CVST (36), PE (36)	None	None	Warfarin (2.5 mg, every day)
**Ⅱ-15**	M	55	23.03	N	-	None	COPD	-
**Ⅲ-18**	F	29	27.47	N	-	None	Thalassemia	-
**Ⅳ-4**	M	16	26.47	N	-	None	None	-

In addition to anticoagulant therapy medications, Ⅱ-7 was receiving irbesartan and hydrochlorothiazide tablets (150 mg/12.5 mg, every day) and aspirin (irregular; the most recent administration of 100 mg occurred 7 days prior) during general blood and coagulation tests, and subsequently rivaroxaban (20 mg, every day) before TGA.

AIS, acute ischemic stroke; BMI, body mass index; COPD, chronic obstructive pulmonary disease; CVST, cerebral venous sinus thrombosis; DVT, deep vein thrombosis; F, female; HL, hyperlipidemia; HT, hypertension; M, male; MVT, mesenteric vein thrombosis; N, no; PE, pulmonary embolism; Y, yes.

### 3.2 Coagulation tests

The coagulation function test results of 9 available family members are shown in Table 2. For patient safety reasons, anticoagulant therapy for the members with a history of VTE (Ⅱ-17, Ⅲ-2, Ⅲ-7, and Ⅲ-9) could not be discontinued during the conduct of coagulation tests. Consequently, their coagulation function assessments were inevitably affected by the anticoagulant medications. Furthermore, the coagulation function of Ⅲ-9 may have been significantly influenced by recent excessive warfarin treatment, while the coagulation function of Ⅲ-18 may have been influenced by childbirth nearly 2 months ago. After excluding the results of Ⅲ-9, the remaining 5 mutation carriers had PT, thrombin time, and PTA values within the normal range, and a slight reduction in aPTT was observed only in Ⅱ-5 and Ⅱ-7. Among these 5 individuals, PC, plasminogen, and AT Ⅲ activity were all within the normal range. However, Ⅱ-5, Ⅱ-7, Ⅱ-17, and Ⅲ-7 exhibited varying degrees of decreased PS activity. It is noteworthy that during the assessment of PS activity, Ⅱ-17 was actively taking rivaroxaban, and Ⅲ-7 was on warfarin therapy, which inevitably affected the results. All individuals were negative for lupus anticoagulant.

**Table 2:** Coagulation function test results.

Items	Ⅱ-5	Ⅱ-7	Ⅱ-15	Ⅱ-17	Ⅲ-2	Ⅲ-7	Ⅲ-9	Ⅲ-18	Ⅳ-4	Reference range
PT (s)	10.50	11.30	10.30	10.90	11.70	12.60	33.20	9.60	10.40	10.5-13.7
aPTT (s)	21.00	23.40	28.10	28.40	29.40	27.60	42.70	27.70	24.70	24.8-33.8
TT (s)	18.50	18.60	17.40	17.60	17.90	18.50	17.40	17.60	17.40	14.0-21.0
PTA (%)	129.00	110.80	134.20	119.40	103.10	88.50	23.10	155.20	131.60	75-135
INR	0.96	1.04	0.94	1.00	1.07	1.16	3.11	0.88	0.95	0.80-1.50
FIB (g/L)	2.31	2.48	2.98	3.32	2.75	2.64	5.37	2.96	3.22	1.8-3.5
D-dimer (mg/L)	0.83	0.75	0.18	0.14	0.15	0.43	0.10	0.25	0.10	0.00-0.55
PS (%)	40	26	76	72	81	49	24	88	88	Male: 77-143 Female: 55-123
PC (%)	124	108	93	81	94	97	56	121	110	70-130
PLG (%)	104	87	90	112	82	112	136	133	107	80-120
AT Ⅲ (%)	111	92	102	82	94	109	121	105	111	80-120
LA	−	−	−	−	−	−	−	−	−	

Ⅱ-5, a mutation carrier, with a history of deep vein thrombosis (DVT), was not receiving any medication at the time of testing. Ⅱ-7, a mutation carrier, with a history of DVT and mesenteric vein thrombosis, was receiving irbesartan and hydrochlorothiazide tablets (150 mg/12.5 mg, every day) and aspirin (irregular; the most recent administration of 100 mg occurred 7 days prior) at the time of testing. Ⅱ-15, a nonmutation carrier, with no venous thromboembolism (VTE) history, was not receiving any medication at the time of testing. Ⅱ-17, a mutation carrier, with a history of DVT and pulmonary embolism (PE), was receiving rivaroxaban (10 mg, every day) at the time of testing. Ⅲ-2, a mutation carrier, with a history of DVT and PE, was receiving rivaroxaban (irregular; the most recent administration of 10 mg occurred 2 days prior) at the time of testing. Ⅲ-7, a mutation carrier, with a history of DVT and PE, was receiving warfarin (1.25 mg, every day) at the time of testing. Ⅲ-9, a mutation carrier, with a history of acute ischemic stroke, cerebral venous sinus thrombosis, and PE, was receiving warfarin (2.5 mg, every day) at the time of testing. Ⅲ-18, a nonmutation carrier, with no VTE history, was not receiving any medication at the time of testing. Ⅳ-4, a nonmutation carrier, with no VTE history, was not receiving any medication at the time of testing. aPTT, activated partial thromboplastin time; AT, antithrombin; FIB, fibrinogen; INR, international standard ratio; LA, lupus anticoagulant; PC, protein C; PLG, plasminogen; PS, protein S; PT, prothrombin time; PTA, prothrombin activity; TT, thrombin time.

### 3.3 *F2* gene analysis

**Figure 2:**
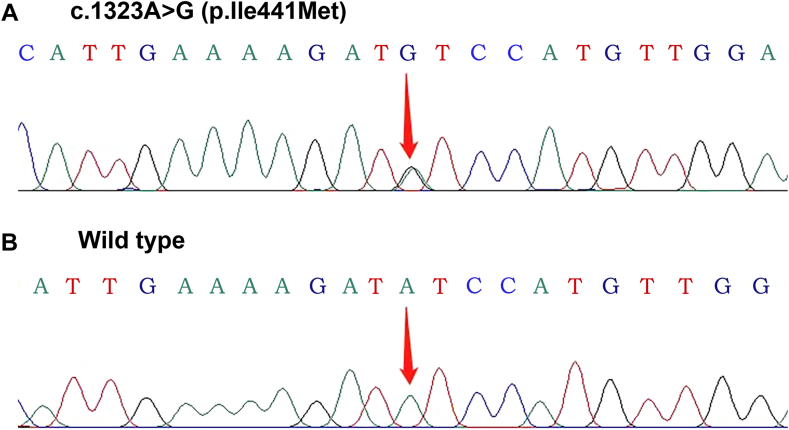
(A) Partial sequencing diagram of *F2* c.1323A>G mutation. (B) Partial sequencing diagram of *F2* wild type.

Genomic DNA sequencing of Ⅲ-9 and Ⅱ-7 revealed a novel heterozygous prothrombin mutation, p.Ile441Met (c.1323A>G; Figure 2). This mutation has not been previously reported, and there are no records of it in ClinVar, Genome Aggregation Database, or Human Gene Mutation Database. Its pathogenicity and underlying mechanism remain unclear. This mutation was also identified in all the other existing family members with a history of VTE (Ⅱ-5, Ⅱ-17, Ⅲ-2, and Ⅲ-7), but was not detected in family members without a history of VTE (Ⅱ-15, Ⅲ-18, and Ⅳ-4). The mutation follows an autosomal dominant pattern, and all carriers of the mutation are heterozygous, contributing to IT within the extended family of Ⅲ-9.

### 3.4 TGA

**Figure 3:**
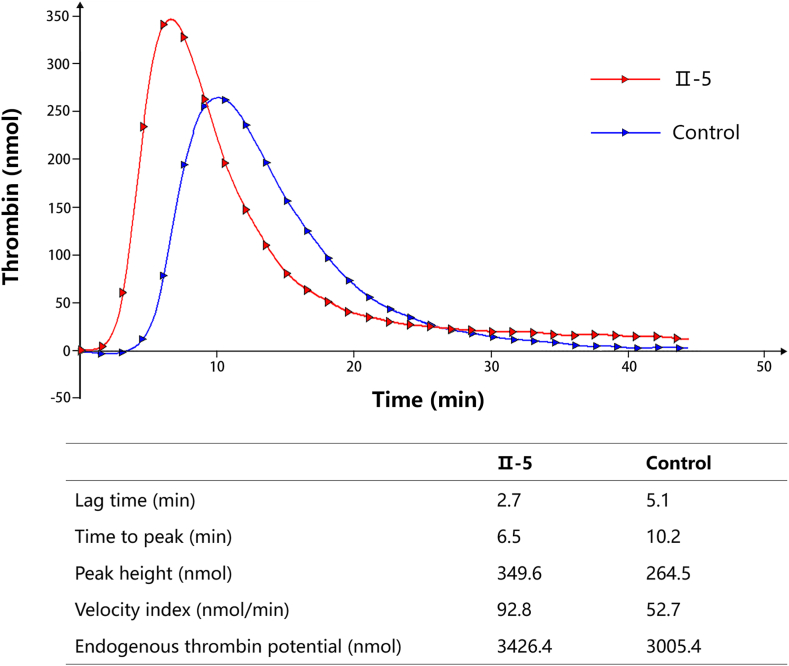
The results of thrombin generation assay.

As other family members carrying the Ile441Met mutation had taken medications affecting coagulation function within the past 3 months, only individual Ⅱ-5 underwent the TGA (Figure 3). Compared with normal controls, Ⅱ-5 exhibited a shortened lag time (2.7 vs 5.1 minutes) and time to peak (6.5 vs 10.2 minutes), alongside an increased peak height (349.6 vs 264.5 nmol) and velocity index (92.8 vs 52.7 nmol/min), resulting in an elevated ETP (3426.4 vs 3005.4 nmol). This result indicates that the coagulation system of mutation carriers is more readily activated and the coagulation process progresses more rapidly, leading to an intensified coagulation response. These findings indicate that the mutation carrier Ⅱ-5 is in a hypercoagulable state, consistent with the prothrombotic risk associated with the Ile441Met mutation.

### 3.5 Protein function prediction

**Figure 4:**
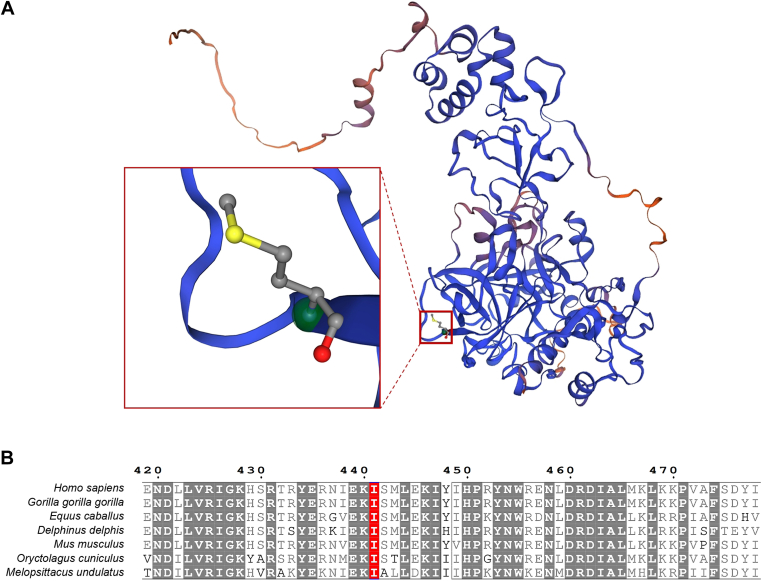
(A) Homology modeling of Ile441Met mutant prothrombin. (B) Conserved analysis of amino acid sequences near Ile441 (marked in red).

SIFT predicted that the substitution of methionine for isoleucine at position 441 would “affect protein function” with a score of 0.00, indicating a significant impact. PolyPhen-2 categorized the mutation as “probably damaging” with a score of 1.000 (specificity: 1.00), suggesting a high likelihood of functional impairment. Similarly, MutationTaster predicted the mutation to be “disease-causing” with a probability value close to 1.00, noting that it might alter protein features and potentially impact splice sites. Conservation analysis across multiple eukaryotic species revealed that the region surrounding the Ile441 residue is highly conserved (Figure 4B), underscoring its critical role in maintaining protein structure and function. Structural modeling using SWISS-MODEL indicated that Ile441 is located within a beta-sheet region, as confirmed by UniProt database annotations (Figure 4A).

## 4 Discussion

In this study, we identified a novel prothrombin mutation, p.Ile441Met (c.1323A>G), in a Han Chinese family with IT. Previous studies of *F2* gene mutations associated with IT have primarily focused on G20210A, while little is known about whether other *F2* variants might also contribute to IT in the Chinese population. In a recent study, Wu et al. [6] screened 347 patients for genetic variations related to thrombosis and hemostasis, identifying 5 heterozygous prothrombin mutations in 12 patients (3.5%): Phe382Ser, Phe382Leu, Asp597Tyr, Arg541Trp, and Arg596Gln. The TGA results revealed a hypercoagulable state in all mutation carriers, with the Arg596Gln mutation exhibiting the highest thrombotic potential [6]. This finding aligns with our study, where the Ile441Met mutation exhibited a similar trend of hypercoagulability.

IT is primarily characterized by various forms of VTE, such as DVT, PE, CVST, mesenteric vein thrombosis, etc. Furthermore, certain types of IT may also manifest as arterial thrombotic events such as AIS and acute coronary syndrome, particularly in cases associated with *F5* Leiden mutation, *F2* G20210A mutation, PS deficiency, and PC deficiency [11]. A large sample multicenter study indicated no significant differences in the anatomical location of VTE caused by different types of IT, with the most common sites ranked as follows: lower-limb DVT (43%-56%), PE (35%-49%), upper-limb DVT (2.6%-4.0%), superficial venous thrombosis (1.7%-3.7%), splanchnic venous thrombosis (0.27%-3.3%), CVST (0%-0.98%), and other locations (0%-1.7%) [1]. This aligns with our findings, where among the 6 mutation carriers, 5 presented with DVT, 4 with PE, and 1 with CVST.

Regarding VTE risk factors, various types of IT, including those resulting from prothrombin mutations, typically manifest as unprovoked VTE, indicating the absence of significant risk factors prior to onset [1]. In our study, only Ⅱ-7 had a significant risk factor for pregnancy, while Ⅱ-15, Ⅱ-17, Ⅲ-2, Ⅲ-7, Ⅲ-9, and Ⅳ-4 had a low relative risk of male sex [10,12]. For patients with significant VTE risk factors, the most common risk factors are immobilization ≥4 days, estrogen use, recent surgery, active cancer, and pregnancy or the postpartum period [1]. Both IT patients and healthy individuals can be affected by these acquired risk factors, but IT patients are more likely to develop VTE when exposed to the same external stimulus due to their genetic hypercoagulability [13]. Therefore, it is essential to conduct VTE risk assessments in IT patients who carry relevant pathogenic genes. Current guidelines suggest that for IT patients without a history of VTE, basic preventive measures such as lower-limb exercises and hydration maintenance are generally sufficient, and preventive anticoagulant treatment is usually not recommended [14,15]. However, when exposed to VTE risk factors, proactive preventive anticoagulant therapy should be undertaken, and in cases where anticoagulation is contraindicated, physical prophylactic measures, such as the use of elastic stockings or foot venous pumps, should be considered [[15], [16], [17]].

Studies have shown that patients with IT caused by *F2* gene mutations often exhibit PT and aPTT values within the normal range [6,18,19], consistent with the findings of our study. As PT and aPTT are the most commonly used clinical indicators for screening coagulation abnormalities, these results emphasize the importance of TGA and genetic testing in individuals with a high suspicion of thrombophilia. Additionally, in our study, 5 of 6 mutation carriers exhibited varying degrees of decreased PS activity. On one hand, this phenomenon may be attributed to the fact that PS, as a vitamin K-dependent protein, has its hepatic synthesis inhibited by warfarin, a vitamin K antagonist [20,21]. On the other hand, excessively elevated levels of thrombin in a hypercoagulable state can indirectly accelerate the consumption of PS, resulting in a decrease in the levels of free PS [22]. However, whether the Ile441Met mutation directly impacts the activity of PS and PC and leads to defects in the PC pathway requires further verification through molecular experiments.

Several online tools and bioinformatics analyses were utilized in this study to evaluate the pathogenicity and potential mechanism of the Ile441Met mutation. Multiple online prediction tools suggested that the Ile441Met mutation exhibits significant pathogenicity, and protein structure analysis indicated that the mutation is located within a beta-sheet region. Beta-sheets are essential components of protein secondary structure, and alterations in this region could disrupt protein folding or interfere with molecular interactions [23]. Based on these findings, it is hypothesized that the Ile441Met mutation affects the structural stability of the beta-sheet and potentially alters interactions with other molecules, such as AT or PC. These changes could impair the regulation of the coagulation process, leading to a hypercoagulable state, as supported by the TGA results showing enhanced thrombin generation. Therefore, the Ile441Met mutation likely contributes to IT through disruption of coagulation regulation and an increased thrombotic risk. However, these hypotheses still need to be further confirmed by *in vitro* experiments.

Some current studies have shown that the molecular mechanisms of prothrombin heterozygous mutations leading to thrombophilia are diverse. One of the most common pathological mechanisms is the impairment of the structure and function of the sodium-binding site of prothrombin due to mutations, including Arg596Leu, Arg596Gln, and Arg596Trp, which subsequently reduces the ability of thrombin to bind to AT, resulting in AT resistance [6,24,25]. This type of mutation typically manifests in TGA results as a reduced thrombin peak but a significantly prolonged decay time, leading to increased ETP values [19,25]. In contrast, our study observed that the TGA results for carriers of the Ile441Met mutation showed a significantly higher thrombin peak compared with normal controls, with no notable change in decay time. Additionally, another study of the Arg541Trp mutation revealed that it reduces the affinity between thrombin and PC, impairing PC activation and causing defects in the PC anticoagulant pathway [26]. Similar to our findings, carriers of the Ile441Met mutation exhibited significantly elevated thrombin peak levels. In addition, the G20210A mutation leads to increased thrombin peak values in TGA results by enhancing the antifibrinolytic pathway dependent on thrombin-activatable fibrinolysis inhibitor [27]. However, unlike the Ile441Met mutation, the TGA results of the G20210A mutation exhibit a delayed peak time, and there is no significant change in the velocity of thrombin generation.

This study has several limitations. First, the analysis was restricted to a single family, limiting the generalizability of our findings to the broader population. Further studies with larger cohorts and diverse populations are needed to validate the pathogenicity of the Ile441Met mutation. Second, for patient safety reasons, only 1 patient underwent the TGA. Although TGA data provided functional insights, studies using molecular modeling or *in vitro* assays are necessary to confirm the effects of TGA on PTA and coagulation pathways. Lastly, anticoagulant therapy in several mutation carriers may have confounded coagulation test results, necessitating caution in interpreting their phenotypic data.

## 5 Conclusion

In conclusion, this study identified a novel heterozygous prothrombin mutation, p.Ile441Met (c.1323A>G), associated with IT in a Han Chinese family. All available family members carrying the mutation have developed VTE, including PE, lower-limb DVT, mesenteric vein thrombosis, and CVST. The TGA demonstrated a hypercoagulable state in mutation carriers, characterized by enhanced thrombin generation and increased ETP. Further studies are needed to validate these findings and investigate the underlying pathogenic mechanisms.
